# Whole-Body Regeneration in Sponges: Diversity, Fine Mechanisms, and Future Prospects

**DOI:** 10.3390/genes12040506

**Published:** 2021-03-29

**Authors:** Alexander Ereskovsky, Ilya E. Borisenko, Fyodor V. Bolshakov, Andrey I. Lavrov

**Affiliations:** 1 Institut Méditerranéen de Biodiversité et d’Ecologie Marine et Continentale (IMBE), Aix Marseille University, CNRS, IRD, Station Marine d’Endoume, Rue de la Batterie des Lions, Avignon University, 13007 Marseille, France; 2 Department of Embryology, Faculty of Biology, Saint-Petersburg State University, 199034 Saint-Petersburg, Russia; i.borisenko@spbu.ru; 3 Evolution of Morphogenesis Laboratory, Koltzov Institute of Developmental Biology of Russian Academy of Sciences, 119334 Moscow, Russia; 4 Pertsov White Sea Biological Station, Biological Faculty, Lomonosov Moscow State University, 119192 Moscow, Russia; fedbolsh@mail.ru (F.V.B.); lavrovai.bio@yandex.ru (A.I.L.)

**Keywords:** whole-body regeneration, Porifera, morphogenesis, transdifferentiation, differentiation, body polarity

## Abstract

While virtually all animals show certain abilities for regeneration after an injury, these abilities vary greatly among metazoans. Porifera (Sponges) is basal metazoans characterized by a wide variety of different regenerative processes, including whole-body regeneration (WBR). Considering phylogenetic position and unique body organization, sponges are highly promising models, as they can shed light on the origin and early evolution of regeneration in general and WBR in particular. The present review summarizes available data on the morphogenetic and cellular mechanisms accompanying different types of WBR in sponges. Sponges show a high diversity of WBR, which principally could be divided into (1) WBR from a body fragment and (2) WBR by aggregation of dissociated cells. Sponges belonging to different phylogenetic clades and even to different species and/or differing in the anatomical structure undergo different morphogeneses after similar operations. A common characteristic feature of WBR in sponges is the instability of the main body axis: a change of the organism polarity is described during all types of WBR. The cellular mechanisms of WBR are different across sponge classes, while cell dedifferentiations and transdifferentiations are involved in regeneration processes in all sponges. Data considering molecular regulation of WBR in sponges are extremely scarce. However, the possibility to achieve various types of WBR ensured by common morphogenetic and cellular basis in a single species makes sponges highly accessible for future comprehensive physiological, biochemical, and molecular studies of regeneration processes.

## 1. Introduction

All organisms tend to maintain their integrity and show abilities to restore after various damages. Such restoration processes occur at different biological levels through dramatically distinct mechanisms and can produce new structures of different similarity to the lost ones. This phenomenon unities as distinct biological processes as reparative regeneration, physiological regeneration, hypertrophy, etc. [1,2].

In this paper, we will consider only post-traumatic restoration processes in multicellular animals, i.e., reparative regeneration. While virtually all animals show certain abilities for regeneration after an injury, these abilities vary greatly among metazoans. Representatives of some phyla have rather limited regenerative abilities, showing imperfect restoration of lost parts even after minor traumas. In contrast, other animals can completely restore their bodies from small body fragments or even suspension of individual cells [3].

The reparative regeneration can occur at various scales: from the restoration of damaged cells, tissues, and organs to the reconstruction of a whole body from a small portion of tissues, i.e., Whole-Body Regeneration (WBR) [2,4,5]. The term WBR implies the ability to reconstruct an animal’s entire body from small fragments of the original organism. The described examples are few and mostly restricted to basal metazoans: sponges [3,6], placozoans [7,8], ctenophores [9], and cnidarians [10,11]. However, WBR is also known in acoels [12], flat worms [13], nemerteans [14,15], annelids [16,17], echinoderms [18,19], hemichordates [20], and colonial botryllid ascidians [5,21,22].

WBR can occur either with retaining the original organism’s polarity and symmetry or through complete re-building of involved tissues, accompanied by de novo formation of body axes. The latter type of WBR was referred to as somatic embryogenesis. The somatic embryogenesis represents the most profound regenerative process in metazoans, occurring only after deep disintegration of the normal tissue organization, loss of polarity, and symmetry in regenerating fragment [1,23,24].

**Figure 1:**
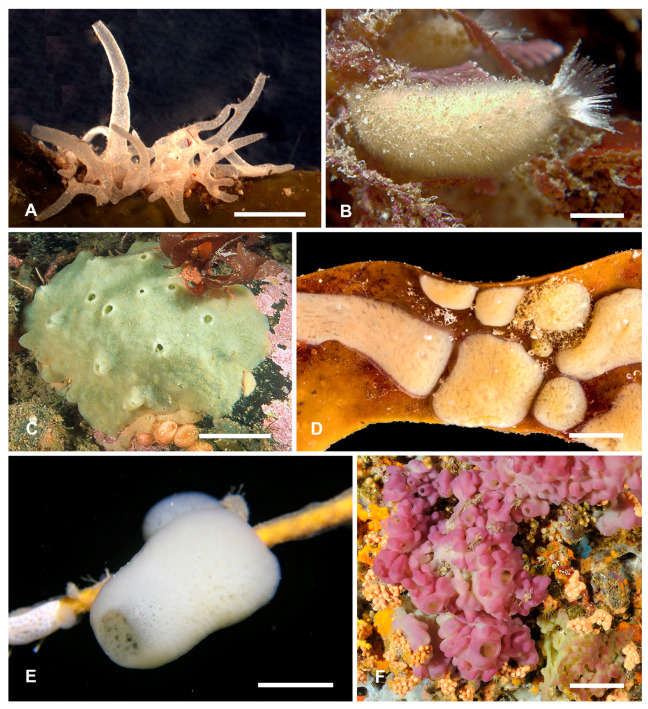
Representatives of different classes of the phylum Porifera *in vivo*. (**A**)—*Leucosolenia variabilis*, Calcarea; (**B**)—*Sycon ciliatum*, Calcarea; (**C**)—*Halichondria panicea*, Demospongiae; (**D**)—*Halisarca dujardinii*, Demospongiae; (**E**)—*Oopsacas minuta*, Hexactinellida; (**F**)—*Oscarella lobularis*, Homoscleromorpha. Scale bars: (**A**)—1 mm; (**B**)—2 mm; (**C**)—5 cm; (**D**)—1 mm; (**E**)—4 mm; (**F**)—5 cm.

Porifera (Sponges) is a basal linage of metazoans [25] characterized by a particular body organization: they lack a gut, muscles, nerves and conventional neuronal signaling systems, organs, and organ systems homologous to Eumetazoa, but have a unique aquiferous system, through which sponges continuously pump water to feed and respire [26]. Adult sponges possess apical-basal polarity, defined by the attachment to substrate at one side and presence of large exhalant openings, oscula, generally at the opposite side. This phylum consists of four classes: Demospongiae, Hexactinellida, Homoscleromorpha, and Calcarea (Figure 1). 

One of the characteristic features of the sponges, distinguishing them from other animals, is the high plasticity of anatomic and tissue structures and cellular differentiation throughout the life cycle. Various differentiated cells of the sponge can transdifferentiate, move and switch functions, depending on the current needs of an organism. Thus, the sponge is constantly in the state of rearrangement of all its structures [27,28,29,30,31]. This “chronic morphogenesis” contributes to the growth, regeneration, movements of the sponge, and reconstruction of somatic tissue after degradation during sexual and asexual reproduction [6,28,29,31,32,33,34].

Representatives of the classes Demospongiae, Homoscleromorpha, and Calcarea are capable of a wide variety of different regenerative processes: healing wounds, regenerating excised body parts, restoring from tissue fragments of various sizes, and restoration during cell reaggregation (for review see: [35,36,37,38]).

Considering phylogenetic position and unique body organization, sponges are highly promising models to study regenerative processes, as they can shed light on the origin and early evolution of regeneration in general and WBR in particular.

In the literature, there is an ingrained opinion that the regenerative abilities of sponges are unlimited. Such ideas emerged after observing the complete restoration of functional sponges from cell suspensions obtained through sponge tissue dissociation. Wilson [39,40] first described this process called reaggregation in marine demosponge *Clathria prolifera* and later on was observed and closely investigated in many different sponge species from classes Demospongiae and Calcarea [3,41]. 

However, the regenerative abilities of sponges are not unlimited. Being a highly complex process, regeneration is affected by innumerous factors, both external and internal. For instance, it was shown that tissue rearrangements associated with sexual and asexual reproduction [31,42,43,44,45,46], as well as environmental factors [47,48], affect the course of various regenerative processes in sponges. As such, it turns out to be difficult to unambiguously assess the regenerative potency of sponges without careful and detailed studies.

To date, reparative regeneration in sponges is much better studied. Morphogenesis, cellular sources, and dynamics of cell proliferation during reparative regeneration of the body wall have been studied at histological and ultrastructural levels in sponges from the classes Demospongiae [37,49,50,51], Homoscleromorpha [36], and Calcarea [38,52,53]. These studies are consistent in denoting transdifferentiation as one of the essential mechanisms of reparative regeneration in sponges. However, further comparative analysis indicates that other mechanisms underlying regeneration are fundamentally different in different clades of sponges. In sponges from the classes Homoscleromorpha and Calcarea, epithelial morphogenesis predominates during regeneration, and the primary cellular sources (pluripotent cells) for the restoration of the lost structures are choanocytes and pinacocytes. At the same time, Epithelial-to-Mesenchymal transformations (EMT) and mesenchymal morphogenesis dominate in demosponge regeneration, and archaeocytes and choanocytes are the primary cellular sources. Moreover, only in demosponges, regeneration is accompanied by the formation of a blastema, which is absent during regeneration in Homoscleromorpha and Calcarea. 

The key difference between WBR and reparative regeneration is the amount of cellular material at the start of the regenerative process. Reparative regeneration initially attracted more attention because it is common to different phyla of animals from lower metazoans to vertebrates. Later on, WBR turned out to be a similarly widespread phenomenon in invertebrates, prompting researchers to search for common features of this process. It is not surprising that WBR in phylogenetically distant groups occurs through different cellular and morphogenetic mechanisms. However, it remains unclear if molecular mechanisms governing WBR are conserved, raising many questions. How conserved is the role of molecular mechanisms, whose participation was described earlier in reparative regeneration? Are all molecular pathways involved in regeneration universal, or do taxon-restricted genes also play key roles in some processes? What molecules govern complex cellular behavior (such as EMT) in the absence of key regulatory proteins that we know? All of these and many other questions outline the role of WBR in modern regenerative biology as a promising model for studying regeneration in invertebrates. These issues are especially acute from an evolutionary perspective, requiring concentrated efforts to study WBR across major metazoan clades, including basal linage: Cnidaria, Placozoa, Ctenophora, and Porifera.

## 2. Whole-Body Regeneration in Sponges

In sponges, there are two principal types of WBR: (1) WBR from a body fragment and (2) sponge restoration in the course of cell reaggregation. In the case of WBR from a body fragment, regeneration could occur either without polarity disruption (regeneration *bona fide*) or with polarity disruption and its subsequent reestablishment (somatic embryogenesis). Cell reaggregation always occurs with polarity disruption through somatic embryogenesis.

The ability for WBR and the mechanisms underlying this process differs not only in sponges belonging to distinct phylogenetic groups but also depends on the organization of the aquiferous system (asconoid, syconoid, or leuconoid). 

### 2.1. WBR from a Body Fragment

WBR in the form of regeneration from a body fragment is very widespread in sponges, regardless of their taxonomic position. Sponge farming, used for obtaining commercial sponges since the early 20th century, is based on this regeneration ability [54,55]. Besides, WBR from a body fragment allows the introduction of the so-called “sandwich method” [56] into experimental researches. The “sandwich preparation” represents a small fragment of sponge tissues tightened between a glass slide and cover glass. After the period restoration, “sandwich preparation” allows direct microscopic observations of living cells’ behavior in sponge tissues, which have resulted in several discoveries in physiology and cell biology of sponges [57,58]. 

#### 2.1.1. WBR after Body Dissection

This type of WBR is close to a typical reparative regeneration known in many other animals, e.g., *Hydra* [10], Platyhelminthes [13], and Asteroides [19]. It is worth noting that WBR after body dissection can occur either with the retention of the parental sponge’s polarity or with complete re-building of involved tissues, i.e., through somatic embryogenesis.

**Figure 2:**
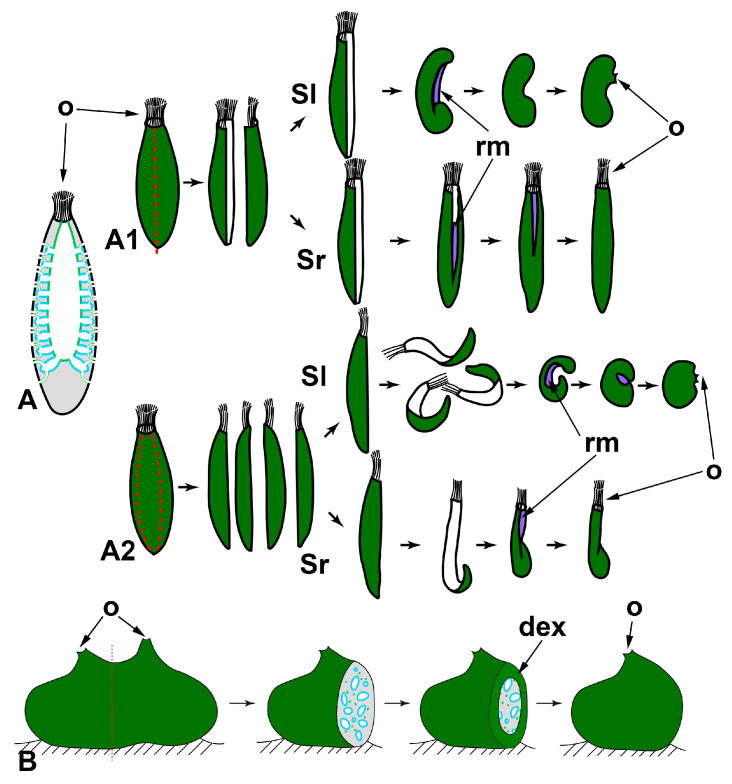
WBR after longitudinal dissections in syconoid (**A**) and leuconoid (**B**) sponges. A1—dissection into halves; A2—dissection into quarters. Sl—*Sycon lingua*, Sr—*Sycon raphanus*. dex—developed exopinacoderm; o—osculum; rm—regenerative membrane. Red dashed line indicates the direction of the dissection; dark green—exopinacoderm; light green—endopinacoderm; blue—choanoderm.

- *Longitudinal dissections* (Figure 2A)

In calcareous sponges, regeneration after the longitudinal dissection was studied in monoscular *Sycon raphanus* with a narrow atrial cavity and polyoscular *Sycon lingua* with a vast atrial cavity. The sponges were dissected into halves or quarters. Each fragment of the sponge body contains all tissues (exo- and endopinacoderm, mesohyl, choanoderm) and parts of essential anatomical elements (numerous radial choanocyte chambers both intact and injured, parts of atrial cavity and osculum). After both types of dissection, a whole functional individual is restored in both species [59,60]. However, regenerative morphogeneses are different in *S. raphanus* and *S. lingua*. In monoscular *S. raphanus*, fragments close up on the atrial side in a baso-apical direction through converging and subsequent coalescence of the wound edges. Quarters can also be partially closed due to the bending of the basal parts in the longitudinal direction. Nevertheless, all fragments in *S. raphanus* retain their original polarity during regeneration, and the oscular region with its typical arrangement of skeletal elements does not undergo anarchization (Figure 2A) [59,60].

In polyoscular *S. lingua*, fragments also close up on the atrial side but through bending in the longitudinal direction. Simultaneously, the apical part of the fragments anarchizes and ceases to function as an osculum. These processes could be considered as signs of loss of original polarity in regenerating fragments. As a result, blindly closed spherical regenerates are formed. Subsequently, they develop a new osculum region at the site of coalescence of wound edges (Figure 2A) [59,61]. 

Besides bending and converging of the wound edges, the closing of regenerating halves and quarters is ensured by the formation of a temporary structure—regenerative membrane (RM). The development and subsequent transformations of RM are well-described at the cellular and ultrastructural levels during WBR after transversal dissection (see below) and reparative regeneration of the body wall in *Leucosolenia variabilis* [38]. In both *Sycon* species, RM develops between converging wound edges of fragments, ensuring fast isolation of the atrial cavity of regenerates [59,61].

Thus, while the developmental mechanisms are essentially the same, different outcomes are possible during WBR after longitudinal dissection in two close *Sycon* species differing in the details of the anatomy (number of oscula, size of the atrial cavity). The development of a new sponge in *S. lingua* is accompanied by the formation of a new apico-basal polarity, while in *S. raphanus,* the original axis is preserved.

Korotkova [6] associated the differences in the WBR of these two species with the degree of their integration (individualization), believing that the integration of the monoscular sponge (*S. raphanus*) is higher than that of the polyoscular one (*S. lingua*). However, the described developmental features could be associated simply with the difference in the rigidity of the skeleton of the investigated species: the skeleton is very strong and rigid in *S. raphanus*, while it is much looser in *S. lingua*, possibly allowing bending of regenerating fragments in the longitudinal direction.

Dissection of polyoscular demosponges into two parts in the apico-basal direction, without separating from the substrate, always leads to incomplete regeneration (Figure 2B). Regeneration occurs through epithelialization of the wound surface, followed by slow restoration of normal organization in the wound area. However, dissected sponges never restore their original shape and size ([54,62,63,64], our unpublished results). The morphogenesis and cell sources ensuring the epithelization and restoration of the normal organization in the wound area are the same as during reparative regeneration of the body wall in demosponges [37,43,50,65,66]. No significant rearrangements of the skeleton or aquiferous system occur in areas remote from the wound (our unpublished results).

- *Transversal sections*

**Figure 3:**
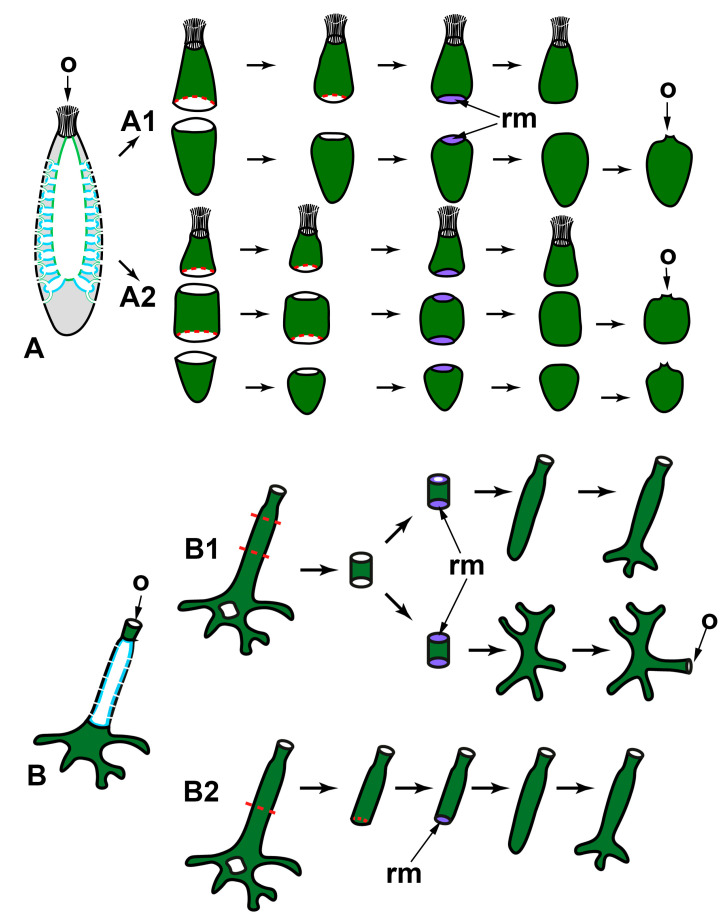
WBR after transversal dissections in syconoid (**A**) and asconoid (**B**) sponges. A1—dissection into two halves of *Sycon* spp.; A2—dissection into three parts of *Sycon* spp.; B1—dissection of the ring from the middle part of the oscular tube of *Leucosolenia* spp.; B2—dissection of the apical part of the oscular tube of *Leucosolenia* spp. o—osculum; rm—regenerative membrane. Red dashed line indicates the direction of the sponge sections; dark green—exopinacoderm; light green—endopinacoderm; blue—choanoderm.

WBR in sponges after transversal dissection of their body (perpendicular to the apico-basal axis) were studied only in calcareous sponges, as many of them have clear apico-basal polarity (Figure 3). Regenerates obtained after the operation are body fragments (rings) retaining all tissues and main structures, as well as their three-dimensional distribution, but lacking the apical and/or basal parts. 

After transversal dissection of monoscular *Sycon raphanus* and *S. ciliatum* and polyoscular *S. lingua* into two, three, or several rings, each ring develops into a new sponge, usually retaining its original polarity (Figure 3A). In *S. raphanus* and *S. ciliatum*, only one osculum necessarily forms on the apical surface of each regenerate [59,60]. In *S. lingua*, while most regenerates also form only one osculum, some of them develop two oscula on their apical surfaces [59]. Additionally, few regenerates of *S. lingua* change their polarity, forming new oscula at the basal parts [59]. 

Rings cut from the middle part of an oscular tube in asconoid *Leucosolenia* spp. also restore a new sponge in the course of WBR. However, while some of the regenerates retain their original polarity, others show change of polarity: in *Leucosolenia complicata* main body axis changes in 12% of regenerates, and in *L. variabilis*, in 50% of regenerates [67]. In this case, new osculum forms as a lateral outgrowth of a regenerate at the late stages of the process (Figure 3B1). Change of the polarity is always accompanied by the reorganization of the whole skeletal structure of regenerates [67]. If rings are cut from the most apical part of an oscular tube and contain an oscular rim (the part of an oscular tube formed by exo- and endopinacocytes), they always restore the intact sponge retaining the original polarity (Figure 3B2). After the wound healing and formation of the new body wall at the basal part of the regenerate, it develops into a sponge retaining the skeletal architecture and the main axis [38,52,68]. 

**Figure 4:**
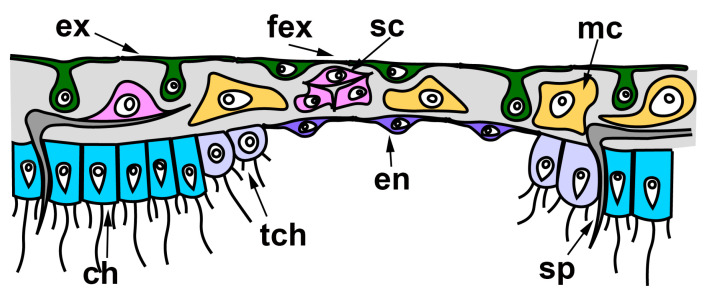
Regenerative membrane in calcareous sponges. ch—choanocyte; en—endopinacocyte of regenerative membrane; ex—intact T-shaped exopinacocyte; fex—flat exopinacocyte of regenerative membrane; mc—cells of mesohyl; sc—sclerocyte; sp—spicule; tch—choanocyte during transdifferentiation.

In all cases described above, regeneration occurs similarly: through the formation of a Regenerating Membrane (RM). The development of an RM is well-described during WBR of oscular tube rings and reparative regeneration of the body wall in *Leucosolenia variabilis* [38]. RM grows from the edges of the wound to its center, closing an opened atrial cavity. The epithelial morphogenesis plays a dominant role in the development of the RM, as it forms due to the convergent spreading and fusion of epithelial layers: the exopinacoderm on the external side of RM and the endopinacoderm—on its internal side. The spreading of the exopinacoderm occurs due to the flattening of T-shaped exopinacocytes, which take a flat shape and consequently increase their area. The endopinacoderm forms and spread through the transdifferentiation of the choanocytes near the wound edges. Similar to the exopinacocytes, the choanocytes obtain a flat shape and increase their area. Additionally, they lose flagella and microvilli collars. This transdifferentiation leads to the massive transformation of flagellar prismatic choanocytes into aflagellar flat endopinacocytes (Figure 4). In *Sycon* species, the inner layer of the RM has a mixed origin: partially, it arises through transdifferentiation of the choanocyte into endopinacocytes, but intact endopinacocytes, lining the atrial cavity and exhalant canals of a sponge, can also contribute to its formation [69,70]. The exopinacoderm and endopinacoderm of the RM are separated by a very thin and almost acellular layer of the mesohyl [38,52]. 

The complete RM closes the wound orifices, isolates the atrial cavity, and transforms into an intact body wall. The mesohyl of the RM obtains an intact structure: the mesohyl cells become more abundant, spaces between them become filled with fibrillar ECM, and sclerocytes begin to synthesize new spicules. Simultaneously, cells of the internal layer of the RM re-differentiated into choanocytes in *Leucosolenia* species but remained endopinacocytes in *Sycon* species. Some exopinacocytes of RM give rise to porocytes through transdifferentiation [38].

Thus, all three layers of the RM (and subsequently of a new body wall) arise through rearrangements of the corresponding layers of the adjacent intact body walls. Cell transdifferentiations accompany these rearrangements: the reversible transdifferentiations of the choanocytes to the endopinacocytes and permanent transdifferentiation of the exopinacocytes to the porocytes. The mesohyl cells do not directly participate in the RM formation and only repopulate the mesohyl [38].

#### 2.1.2. WBR from Small Body Fragments

Small fragments of the body of sponges, regardless of the type of aquiferous system, demonstrate the ability to restore a new sponge in the overwhelming majority of cases. WBR from small body fragments occurs as somatic embryogenesis in both Calcarea and Demospongiae. The principal characteristic of this type of WBR is a profound reconstruction of regenerating tissues. Fragments always lose the initial (characteristic for parental sponge) polarity, undergo a stage of radialization, and finally develop a new apico-basal axis.

**Figure 5:**
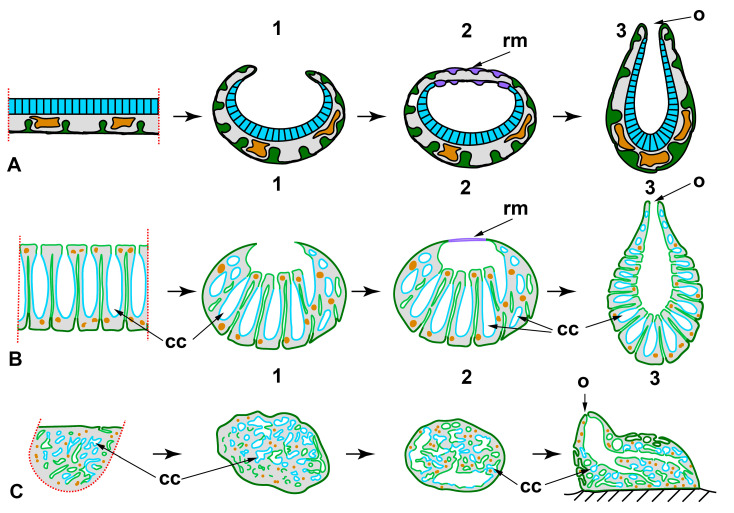
WBR from small body fragments. (**A**)—asconoid calcareous sponge *Leucosolenia* spp.; (**B**)—syconoid calcareous sponge *Sycon* sp.; (**C**)—leuconoid demosponge. Stages of regeneration: 1—epithelialization of the wound surfaces and loss of original symmetry; 2—radialization of a fragment; 3—the restoration of the aquiferous system and formation of an osculum. cc—choanocyte chambers; o—osculum; rm—regenerative membrane. Red dashed line indicates the direction of the sponge sections; dark green—exopinacoderm; light green—endopinacoderm; blue—choanoderm; brown—amoeboid cells of mesohyl.

Excised fragments usually contain both ectosomal and endosomal regions with all essential elements: exopinacoderm, parts of the aquiferous system (canals and choanocyte chambers), mesohyl with appropriate cell composition, ECM, and skeletal elements. However, the exopinacoderm is located only at one (ectosomal) side of a regenerate, while the lateral and basal sides have no exopinacoderm, i.e., represent vast wound surfaces (Figure 5). 

WBR of small fragments proceeds through the following main stages: epithelialization of the wound surfaces, deep disintegration of the aquiferous system elements, cell dedifferentiation, loss of original symmetry and radialization of a fragment, attachment to the substrate, a transition from radial to apico-basal symmetry, restoration of the aquiferous system, and formation of an osculum. 

Small fragments (about 2 × 2 mm) cut from the body wall of asconoid calcareous sponge *Leucosolenia* spp. have an organization identical to the intact body wall. Fragments are slightly concave towards the side of the former atrial cavity. They are lined with the exopinacoderm at the outer side and with the choanoderm—at the atrial side [68,71,72].

At the initial stages of regeneration, the choanoderm near the wounds is temporarily anarchized, and choanocytes dedifferentiate: they lose the collar and flagellum, become rounded, and stop food capturing. Initially flat fragments take a cup shape, bending to the choanoderm (atrial) side. Then RM develops at the edges of a fragment and gradually grows in a centripetal direction, enclosing an opening of a cup-shaped fragment. This process results in the formation of closed spherical regenerate with an atrial cavity. During further regeneration, fragments lose their original polarity and rearrange, i.e., tissue of a fragment per se transforms into the basal parts (future cormus) of the developing sponge, while RM gives rise to a new oscular tube ([72], our unpublished data) (Figure 5A).

One of the clear markers of the apico-basal polarity of the oscular tube in *Leucosolenia* is the organization of its skeleton. While the skeleton of oscular tubes is predominantly formed by an organized net of tri- and tetractines with unpaired actines oriented parallel to the main oscular axis, the skeleton of the cormus tubes is mostly disordered. During the formation of the closed spherical regenerate, the original organized arrangement of the skeleton is disturbed and becomes disordered. Subsequently, the skeleton obtains its typical parallel orientation in forming an oscular tube (our unpublished data).

In the syconoid calcareous sponges, *Sycon ciliatum* and *S. lingua*, small body fragments also develop into a new functional sponge [59,69]. Initially, fragments are more or less flat, covered by the exopinacoderm at the outer side and by endopinacoderm—at the atrial side. The lateral sides of fragments represent vast wound surfaces. Fragments contain intact and damaged elements of the aquiferous system—canals and radial choanocyte chambers. Intact aquiferous system elements are located in the inner parts of fragments, while damaged ones lay near wound surfaces. The further regeneration process is accompanied by a deep reconstruction of the aquiferous system of a fragment (Figure 5B). 

The early stages of the *Sycon* body wall fragment regeneration are similar to the regeneration of the *Leucosolenia* body wall fragments: fragments bend to the atrial side and obtain a cup shape. Simultaneously, wound surfaces at lateral sides of the fragments are epithelized by exo- and endopinacocytes. Then RM develops from the elevated edges of the cup-shaped fragments, and grows in a centripetal direction, forming closed regenerates with a new atrial cavity [59,69]. Closed spherical regenerates gradually transform into functional sponges: similar to the development of *Leucosolenia* fragments, tissues of a *Sycon* fragment per se give rise to the basal parts of the developing sponge, while the apical part with a new osculum develops from RM. Described WBR of fragments is accompanied by an intensive reconstruction of their aquiferous system, including the disintegration of some canals and chambers, formation of new canals and chambers, and reconnection of aquiferous system elements. This process leads to temporary ceasing of water pumping and is especially active near the wound surfaces [59,69].

Recent experiments with syconoid calcareous sponge *Sycettusa hastifera* showed that fragments smaller than 1 mm undergo profound tissue reorganization and transform into epithelized multicellular aggregates, primmorphs [53]. Primmorphs are one of the principal stages of WBR during sponge cell reaggregation. Consequently, further development of primmorphs and reconstruction of intact sponges in *S. hastifera* occurs similar to such processes during cell reaggregation [53] (see Section 2.2). 

Small cubic body fragments cut from the body of demosponges with a leuconoid organization also show abilities to develop into intact sponges [51,56,62,65,73,74,75,76,77,78] (Figure 5C). These fragments can include both ectosome and endosome [51,56,62,65,73,74,75,76,77,78] or only part of the endosome [62,65,77,78,79]. In both cases, the lateral sides of fragments represent vast wound surfaces with naked mesohyl and dissected elements of the aquiferous system. The inner parts of fragments contain intact elements of the aquiferous system. 

Immediately after a surgical operation, aquiferous canals and choanocyte chambers in the wound area begin to disintegrate. The endopinacocytes and choanocytes lose contact with adjacent cells in their epithelial layers and change shape from trapeziform (choanocytes) and flat (endopinacocytes) to spherical or amoeboid. These dedifferentiated cells intermix with the mesohyl cell population. The non-secreting cells of the mesohyl (archaeocytes, lophocytes, dedifferentiated choanocytes, and endopinacocytes) actively phagocytize cell debris, symbiotic and invasive microbes. The same processes occur in the wound area during reparative regeneration in demosponges [37,50].

Subsequently, accumulations of cells of different origins (dedifferentiated choanocytes, archaeocytes) appear under the wound surfaces. Epithelization of the wound surfaces occurs through mesenchymal-to-epithelial transformation (MET): cells of the outermost layer flatten and, together with intact exopinacocytes, form a new exopinacoderm of developing sponge fragment. Epithelized fragments lose their initial polarity, obtain a more or less spherical shape, undergoing radialization. This process is accompanied by deep internal reorganization of fragments, expressing in a redistribution of archaeocytes and dedifferentiated cells, anarchization of a part of the choanocyte chambers and canals, and change in the orientation of some canals. At this stage, canals and choanocyte chambers are partially disconnected from each other, and consequently, the aquiferous system temporarily ceases its filtration activity. After attachment to the substrate, fragments undergo a further transformation, associated with the spreading over the substrate and development of the basopinacoderm. Attachment to the substrate promotes the formation of a new apico-basal axis and further reorganization in the internal parts of developing fragments. This reorganization includes redistribution of fragments of old elements of the aquiferous system, as well as the formation of new ones. Development of new choanocyte chambers and canals occurs due to uniting of archaeocytes and dedifferentiated choanocytes into clusters with their subsequent differentiation into choanocytes or endopinocytes. Thus, these processes occur through MET. The final formation of a functional aquiferous system with a new osculum occurs only at the latest period of regeneration when the definitive apico-basal orientation of canals and layers of the body are completely established [65,74,75] (Figure 5C).

Regenerating body fragments of *Chondrosia reniformis* was used for the first study of molecular mechanisms involved in WBR in sponges. In particular, the involvement of TGF-β signaling in the early stages of WBR (up to complete epithelization of the wound surfaces) was studied. It was shown that three TGF ligands are actively expressed during the first 48 hours post-operation and possibly involved in stem cell maintenance. The other two TGF ligands are upregulated during differentiation of new exopinacocytes and are considered as pro-differentiating factors [51].

Various factors can influence the progression and outcome of WBR from body fragments in demosponges. In sponges with a well-developed cortical layer of the body, the restoration processes and their mechanism are strongly dependent on the morphogenetic potential of the cortical layer and endosome. For example, in *C. reniformis*, fragments containing cortex and endosome or only endosome restore normally organized sponges, while fragments lacking the endosome degenerate after obtaining spherical shape [51,77,79]. On the contrary, the successful intact sponge restoration is ensured by the cortical layer in *Tethya aurantium* [62,65,78]. Archaeocytes and lophocytes, concentrated mainly in the cortical layer, take part in the epithelialization of wound surfaces in this sponge. The subsequent development of a new sponge is accompanied by the reorganization of the aquiferous system and, under certain conditions, the rejection of a part of the mesohyl tissues. Fragments of *T. aurantium* containing only the endosome are fail to restore intact organization and gradually degenerate. The author believes that the inability of endosome fragments to develop into a new sponge is associated with the shortage of archaeocytes and lophocytes necessary for epithelialization in this area of the sponge [62,65,78]

The difference in the formative potencies of the similar region in the body of *T. aurantium* and *C. reniformis* occurs due to the different distribution of pluripotent cells involved in the regeneration process: the archaeocytes are located mainly in the endosome in *C. reniformis* [33], while in *T. aurantium* they are found in large numbers in the cortex, where they serve as the source for the formation of external buds [65].

The stage of the reproductive cycle of a sponge used for an experiment strongly affects the regenerative process. The WBR from small fragments in *Halichondria panicea* proceeds most successfully, ending with functional sponge restoration, during the growth period and at the beginning of the oogenesis [43,44]. In contrast, during the late gametogenesis and embryogenesis, body fragments are predominantly incapable of complete sponge restoration and die during the first 20 days of cultivation [44]. Sexual reproduction in sponges is associated with deep tissue reconstruction and, in particular, with committing of pluripotent cells (archaeocytes and choanocytes) for differentiation into gametes or various supportive cells [80]. These rearrangements of tissues consequently affect the structure and morphogenetic potencies of regenerating fragments, decreasing the number of available pluripotent cells involved in the regeneration process. 

### 2.2. WBR by Aggregation of Dissociated Cells

The sponge cell reaggregation represents one of the most outstanding examples of WBR across animals. During this process, a new functional individual is reconstructed from a cell suspension through the aggregation of individual cells and subsequent development of multicellular aggregates. This striking phenomenon has attracted researchers for more than 100 years after the work of Wilson [39], making cell reaggregation the most studied type of WBR in sponges.

**Figure 6:**
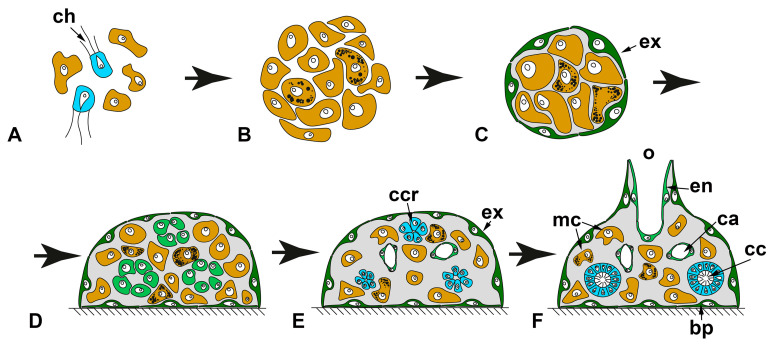
WBR after tissue dissociation (cell reaggregation). (**A**)—cell suspension, consisting of dedifferentiated cells of amoebocyte morphotype and choanocytes; (**B**)—primary multicellular aggregate; (**C**)—primmorph, epithelized aggregate covered with continuous exopinacoderm; (**D**,**E**)—primmorphs with developing aquiferous system; (**F**)—reconstructed functional sponge. bp—basopinacocytes; ch—choanocyte; ca—canals of an aquiferous system; cc—choanocyte chamber; ccr—choanocyte chamber rudiments; en—endopinacocyte; ex—exopinacocyte; mc—cells of mesohyl; o—osculum. Dark green—exopinacoderm; light green—endopinacoderm; blue—choanoderm; brown—amoeboid cells of mesohyl.

For obtaining a cell suspension consisting of individual cells, the sponge tissues are dissociated by chemical [81,82] or mechanical methods [39,41,83]. Such a harsh procedure leads to a complete disruption of tissue integrity, intercellular interactions, and a system of intercellular communication that instructs each cell about spatial body organization in conditions of “chronic morphogenesis.” Further development from cell suspension is full de novo formation of an individual representing a promising model system to study somatic embryogenesis. Sponge cell reaggregation relies on several principle processes: (1) coalescence of cells, leading to the formation of multicellular aggregates and restoration of intercellular interactions, (2) epithelization of the aggregates’ external surfaces, ensuring isolation of their internal milieu, and (3) gradual development of an aquiferous system and other anatomical structures, underlaid by probable re-establishment of positional information and determination of new axes and polarity (Figure 6). The majority of studies on cell reaggregation are made in demosponges, but less information is also available for calcareous sponges (eviewed in 3), and a single preliminary study is done in homoscleromorphs [84].

In all studied species, isolated cells in a suspension obtain a spherical shape shortly after tissue dissociation. Choanocytes retain collars of microvilli and flagella at this stage. The only activity of the cells at this stage is a formation of pseudopodia, and this process usually begins already in suspension but becomes more active after cells reach a substrate [39,41,45,85,86,87,88,89,90,91,92,93,94,95,96,97]. The cells of demosponges usually form filopodia and lobopodia [41,45], while cells of calcareous sponges’ form lamellipodia and scleropodia (long thin cell extension with well-developed actin core; described only in calcareous sponges) [95,96,98]. 

Cell suspensions obtained after tissue dissociation should virtually contain all cell types characteristic for a particular sponge. However, a very limited number of morphologically distinct cell types are found in suspensions: only 3–5 morphotypes, depending on sponge species. The only morphotypes that are found in cell suspensions of every species are amoebocytes (archaeocytes, nucleolated and anucleolated amoebocytes), the most abundant cell morphotype in a suspension, and choanocytes, easily distinguished by the presence of collar and flagellum [39,41,45,83,85,86,87,89,90,91,92,93,94,95,96,97,99,100]. The mass dedifferentiation probably occurs shortly after tissue dissociation, and during it, the majority of cells lose their morphological traits, obtaining morphotype of amoebocytes [41,97,101]. 

The pseudopodial activity of cells in a suspension is the primary driver of cell reaggregation. Cells of amoebocyte morphotype are usually the most active and motile cells, while other cells could be immotile or show limited motility [86,89,90,91,92,93,102]. Apparently, the motility of cells and their occasional contacts gradually lead to the formation of the first multicellular aggregates [27,39,41,45,86,87,89,90,91,94,99,100]. 

Another mechanism of the reaggregation is described for some demosponges: cells in a suspension also form pseudopodia but do not show any motility. Instead, they use the pseudopodia to survey the space around them. In the case of contact between pseudopodia of cells, these cells are pulled to each other forming an aggregate [41,45,103]. For freshwater sponge *Ephydatia fluviatilis,* a mixed mechanism of cell reaggregation is described [91,94]. 

Cells in multicellular aggregates retain their pseudopodial activity, and small aggregates sometimes can creep over a substrate due to it. The further growth of the aggregates occurs through the incorporation of free cells from a suspension or through merging with other aggregates [27,41,45,86,87,89,90,91,94].

At the molecular level, reaggregation of sponge cells occurs with the help of an extracellular proteoglycan-like macromolecular complex, called aggregation factor [104,105]. The aggregation factor comes into solution during the dissociation of sponge tissues. Subsequently, it serves as a bridge, adhering cells to each other through interaction with specific transmembrane receptors [81,105,106]. The aggregation factor is coded by a family of highly polymorphic genes, which transcripts acquire additional diversity with RNA editing. Besides, at least in demosponges, these proteins provide allorecognition properties to each sponge specimen forming a self-nonself recognition system [107].

The gradual reaggregation of cells results in the formation of the primary multicellular aggregates. These aggregates have an irregular shape and poor cell composition similar to the suspension. Primary aggregates represent a loose intermix of cells, which have an amoeboid or spherical shape. Additionally, aggregates can include spicules and their fragments, collagen and spongin fibers, symbiotic bacteria, and algae from parental sponge tissues. This stage of reaggregation is marked by the absence of active morphogenetic processes [41,45,47,82,92,93,94,108,109,110].

After some time, primary aggregates start their transformation into primmorphs—epithelized aggregates [41,45,47,82,84,87,92,93,94,101,108,109,110,111,112,113]. During the transformation, the structure of aggregates changes, mainly in the surface region. The surface cells progressively flatten and transdifferentiate into exopinacocytes. Both cells of amoebocyte and choanocyte morphotypes participate in the epithelization. During the transdifferentiation, choanocytes resorb their collars and flagella [41,45,47,92,93,101,110,111,112,114]. In demosponges, the epithelization occurs through a mesenchymal–epithelial transition (MET): individual surface cells separately transdifferentiate into exopinacocytes and then form contacts with each other, reestablishing continuous exopinacoderm [41,110,114]. In addition to MET [101], calcareous sponges utilize epithelial morphogenesis to form the exopinacoderm. In such cases, a joined group of choanocytes on an aggregate’s surface simultaneously flattens and transdifferentiate into exopinacocytes without breaking their intercellular contacts [114]. The rearrangements in the inner part of the aggregates during their transformation into primmorphs are usually accompanied by the tightening of the cell package [41,82,92,93,108]. The synthesis and change of distribution of ECM fibers can also occur during primmorph formation in some species [92,93,115]. 

The further progressive development of the primmorphs into functional sponges is associated with the formation of main anatomical structures: aquiferous system, skeleton, and mesohyl [39,40,41,45,85,88,92,93,94,100,108,109,110,112]. The restoration of the aquiferous system is the most obvious process during this period and usually attracts the attention of researchers. In most studied demosponges, both choanocyte chambers and canals form due to the active migration of cells and their subsequent transdifferentiation into endopinacocytes and choanocytes through MET. During choanocyte chambers’ development, cells move to each other forming spherical groups—choanocyte chamber rudiments looking like dense cellular rosettes. Further development of rudiments includes expansion of their internal lumen, differentiation of cells into the choanocytes, and establishing connections with canals of the aquiferous system [41,87,92,93,94,108,110]. Canal develop by the opposite process: cells move away from each other, leaving free space in-between them. This space becomes the lumen of the future canal, while cells delimiting it transdifferentiate into endopinacocytes [41,45,88,94]. 

However, for some demosponges, other mechanisms of aquiferous system development are described. In freshwater sponges, *Ephydatia fluviatilis* and *Spongilla lacustris*, choanocyte chambers restoration involve cell proliferation through which the number of choanoblasts in chamber rudiments increases [94,116]. In two marine demosponges, *Halisarca dujardinii* and *Halichondria panicea*, which can form dense multicellular aggregates where cell migrations are probably hampered, the aquiferous system canals are developed through the controlled death of some cells. In this case, the space forming in the place of disintegrating cells is used to build a new canal. Interestingly, the described mechanism is used the inner parts of dense aggregates, while in their outer parts where cell migrations are possible, canals develop through the divergence of cells [45,92,93]. Controlled cell death tends to play a central role in the development of the aquiferous system elements in few studied calcarean species [85,92,93,117]. In contrast to aquiferous system development, the restoration of skeleton and mesohyl remain almost unstudied. 

At the final stage of the reaggregation, primmorphs obtain pores and oscular tubes and start active filtering. From this moment, each aggregate transforms into a small functional sponge with all essential elements. However, reconstructed sponges have a simpler aquiferous system and show the organization of the rhagon in demosponges or olynthus in calcareous sponges [39,40,41,45,85,88,92,93,94,100,108,109,110,112]. 

Another essential process occurring during reaggregation is a restoration of the cellular composition characteristic for intact sponge tissues. If primary aggregates are principally built from choanocytes and cells with amoebocyte morphotype of heterogeneous origin (dedifferentiated cells, i.e., exo- and endopinacocytes, sclerocytes, etc. and amoebocytes/archaeocytes of mesohyl), how are missing cell types restored and from what cell sources? Unfortunately, we have extremely limited data about this process. Almost all we know is that exopinacocytes arise from both choanocytes and cells of amoebocyte morphotype in Demospongiae and Calcarea [41,45,47,92,93,101,110,111,112,114]. Cell sources for the restoration of new cell types during the progressive development of primmorphs and reconstruction of functional sponges remain mostly unknown. Few experimental data obtained from reaggregation in freshwater sponge *E. fluviatilis* point to nucleolated amoebocytes/archaeocytes (depending on the terminology used by different authors) as the main source for all other cell types. Aggregates built exclusively from these cells are able for progressive development and intact sponge reconstruction, while aggregates devoid of these cell types fail to do so [112,118,119,120,121]. 

The only comprehensive study of the molecular mechanisms underpinning cell reaggregation was done in calcareous sponge *Sycon ciliatum* through comparative transcriptomic analysis [101]. More than 9000 genes are differentially expressed during cell reaggregation. Among these genes are core components of the Wnt, Tgfβ, Notch, and Hedgehog signaling pathways, genes of major eumetazoan cell-adhesion families, cell-surface receptors, cytoplasmic linkers, extracellular-matrix proteins, major cell death, or apoptotic-associated pathways. Intriguingly, approximately half of the genes expressed during cell reaggregation are also used during post-larval development. The principal component analysis demonstrates that patterns of differentially expressed genes in regenerating and developing samples converge over time. Thus, late stages of regeneration and development are more similar by a set of transcripts than early ones. This fact indicates that reconstruction of the intact individual through cell reaggregation in *S. ciliatum* utilizes a set of genes similar to the one used during the normal development of this sponge [101].

Despite a widespread opinion that every sponge species can reconstruct functional individuals during cell reaggregation, such ability seems to be more limited: from more than 40 studied species, only 11 can reconstruct functional sponge [3,41,101,108,110]. It is currently unclear what differences are between sponges that are able and unable to reconstruct functional individuals during cell reaggregation. However, experimental data from former species shed some light on the factors that can constrain the progressive development of multicellular aggregates.

The cell-type composition of suspension affects the reaggregation process. Density gradient centrifugation of cell suspension of freshwater sponge *E. fluviatilis* allows obtaining several cell fractions differing in cell-type composition: (1) archaeocyte fraction, (2) mixed cell fraction 1 (containing all cell types, except archaeocytes), (3) choanocyte-enriched fraction, (4) mixed cell fraction 2 (containing all cell types, except archaeocytes and choanocytes). Subsequent experiments show substantial differences in the reaggregation process among these fractions. As was mentioned above, only aggregates obtained from archaeocyte fraction are able for progressive development and intact sponge reconstruction. Aggregates obtained from mixed cell fraction 1 and choanocyte-enriched fraction reach only stages of primmorphs and primary multicellular aggregates, respectively. Cells from mixed cell fraction 2 are unable to reaggregate at all [112,120,121]. Similar results were obtained for marine demosponge *Hymeniacidon perleve* [122]. 

The morphogenetic potencies of multicellular aggregates are also dependent on the size of an aggregate. Some researchers mention that aggregates are unable for progressive development if they are smaller than some critical size: 1000 cells in *E. fluviatilis* [99,116] and 2000 cells in *Clathria prolifera* [88,111], 100 µm in *Sycon lingua* [92,93]. The detailed study of the morphogenetic potencies of multicellular aggregates, depending on their size, showed that only aggregates larger than 200 μm in diameter are able for progressive development in *E. fluviatilis*. Smaller aggregates (50–150 μm in diameter) either are unable for progressive development at all or have very limited morphogenetic potencies. In some cases, small aggregates undergo epithelialization, the formation of a few small choanocyte chambers and canals, but with a marked delay in comparison with larger aggregates. However, small aggregates never reconstruct a complete aquiferous system. Similar results are obtained for aggregates, consisting only of nucleolated amoebocytes: progressive development occurred only in aggregates larger than 250 μm [35,119,123]. 

Finally, various intrinsic and extrinsic factors can affect the physiological state of sponges used for obtaining a cell suspension, which in turn can influence the further reaggregation process. Among extrinsic factors are adverse environmental conditions [31,48] or prolong laboratory cultivation before the dissociation procedure [47]. Intrinsic factors are associated with various somatic tissue rearrangements, occurring, for example, during sexual [31,45] or asexual reproduction [94] and changing the proportion of cell types important for the subsequent reaggregation process (e.g., archaeocytes and choanocytes).

## 3. Conclusions and Future Directions 

If we take a look at the types of regeneration across multicellular animals, we find that representatives of many metazoan phyla show capabilities for various types of WBR:

- WBR after longitudinal dissections is characteristic for ctenophores [9,124], Placozoa [8], *Hydra* [125], planarians [13];
- WBR after transversal dissections is known for ctenophores [124], cnidarians [11,125,126], acoels [12], planarians [13], different spiralians [127], echinoderms [128,129], ascidians [130];
- WBR from small body fragments with polarity disruption is found in *Hydra* [131], planarians [13], colonial ascidians [5];
- WBR by reaggregation of dissociated cells described in placozoans [7], and *Hydra* [132,133].

Animals capable of various WBRs are found in all basal metazoan clades, including sponges, ctenophores, placozoans, and cnidarians. Thus, potentially, WBR could be already present in the last common ancestor of Metazoa and be regarded as an ancestral feature that has been lost from most animal lineages [134,135,136]. 

A fairly high diversity of WBR in sponges could be initially classified, according to the operation type:

1. WBR from a body fragment;

1.1. WBR after body dissection; 

1.1.1. Longitudinal dissections;

1.1.2. Transversal sections;

1.2. WBR from small body fragments;

2. WBR by aggregation of dissociated cells.

**Figure 7:**
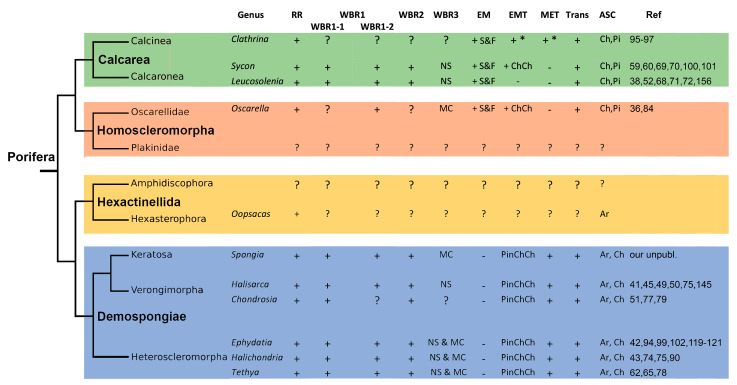
Phylogenetic distribution of different types of regeneration, morphogenesis during regeneration, and adult stem cells across Porifera. RR—reparative regeneration; WBR—whole-body regeneration; WBR1—WBR after dissection; WBR1-1—Longitudinal dissection; WBR1-2—Transversal dissection; WBR2—WBR from small body fragment; WBR3—dissociated cells aggregation (Ns—new functional sponge; MC—multicellular aggregates of various structure); EM—epithelial morphogenesis (S—spreading, F—fusion); EMT—epithelial-mesenchymal transition (ChCh—choanocyte chambers disintegration, Pin—pinacoderm disintegration); MET—mesenchymal-to-epithelial transition; Trans—transdifferentiation; ASC—adult stem cells (Ar—archaeocytes, Ch—choanocytes, Pi—pinacocytes); + presence; − absence; ?—no data. *—porocytes during reparative regeneration.

Despite the main stages of WBR are the same, sponges belonging to different phylogenetic clades and even to different species and/or differing in the anatomical structure undergo different morphogeneses after similar operations (Figure 7). For example, after longitudinal dissection, the restoration of a functional sponge occurs distinctly in calcareous sponges and demosponges. In calcareous sponges, regeneration proceeds through the coalescence of wound edges with the formation of a specific temporary structure, a regenerative membrane. In contrast, the same process involves only the epithelization of the wound surface through a mesenchymal-epithelial transition in demosponges (Figure 2).

Differences in the regenerative mechanisms in closely related species are noticeable during WBR after longitudinal dissection in *Sycon* spp.: while in *S. raphanus*, fragments close up through gradual converging and coalescence of the wound edges, in *S. lingua*, and fragments close up through bending of the whole fragment in the longitudinal direction. Consequently, *S. raphanus* fragments retain their original polarity during regeneration, whereas fragments of *S. lingua* lose the original polarity and reestablish a new one (Figure 2A1,A2) [59,61].

Demosponges with a well-developed cortical layer give a good example of how anatomical structure can influence the regenerative process when a successful regeneration requires the presence of a specific layer in a fragment: the endosome in *Chondrosia reniformis* and the ectosome in *Tethya aurantium* [62,65,77,78].

It is worth noting that despite a wide variety of different regenerative processes in general and WBR in particular, the regenerative abilities of sponges are not unlimited. In the majority of studied species, cells in suspension either do not aggregate at all, or the development of the multicellular aggregates stop after their epithelialization [41,108,137]. Sometimes sponges fail to completely restore even smaller injuries, e.g., superficial wounds in *C. reniformis* [79]. 

Thus, it is incorrect to generally consider WBR mechanisms in sponges. In each specific case, the phylogenetic position of a studied species, its anatomical structure, physiological stage, and the type of operation should be analyzed. Additionally, the distribution of WBR abilities across sponges should be carefully studied. At the moment, there are very few studies of WBR conducted using the same technique on sponges from distant phylogenetic groups or differing in the anatomical organization. Only such scrutiny will allow the conducting of comparative studies and performing analysis of the WBR phenomenon in sponges correctly.

The most specific trait of WBR in sponges is the phenomenon of the instability of the main body axis, defining organism polarity. The polarity change is described during all types of WBR in sponges. The polarity change is facultative in WBR after body dissection but seems to be obligatory in WBR from small body fragments and during cell reaggregation. Intriguingly, the fate of the main body axis is different even in the closely related species after the same surgical operation: after transversal dissection of the oscular tube in *Leucosolenia complicata* and *L. variabilis*. The main body axis changes in 12% of regenerates of the former species, and 50% of regenerates of the latter [67]. Likewise, regenerates of *Sycon raphanus* preserve the original apical-basal axis after longitudinal dissection, while regenerates of *S. lingua* forms a new axis after the same operation [69]. In the similar WBR of other metazoans, the main axes (anterio-posterior or apical-basal) are always preserved. For example, the inheritance of polarity is evident in excised tissues of *Hydra* [131] or planarians [13], which regenerate along the direction of the original body axis. 

WBRs in which the morphological axes of a new organism are reestablished de novo were referred to as somatic embryogenesis [24,138]. According to Korotkova and Tokin [138], somatic embryogenesis can be defined not only by the size of the body fragment and the number of cells that take part in the development of an organism but also by types of involved morphogeneses. Regeneration processes can be affected by different factors, which cause a weakening or disturbance of organism integration mechanisms. The formation of new axes during somatic embryogenesis reflects such weakening and disturbance of integrative systems. This, in turn, causes degradation of tissue and organ structure and formation of accumulations of cells of various origins, from which the rudiment of a new organism develops. Subsequently, new morphological axes and symmetry characteristics of this animal are formed in the rudiment. 

The molecular toolbox underlying axis formation during regeneration is shared among different animals, with a central role for the Wnt signaling pathway [139,140,141,142]. At first glance, sponges show a distinct type of apico-basal polarity, when one part of the body is attached to the substrate, while the opposite bears oscula [143]. Such an organization is found in Calcarea, Demospongiae, and Homoscleromorpha. We know that Wnt ligands differentially express in oscula of calcarean *Sycon* and demosponge *Halisarca* [144,145]. Moreover, it has been shown by the transplantation of oscula that ectopic osculum causes the restructuring of the entire aquiferous system; and pharmacological agents that mimic the active Wnt cascade have a similar effect [146]. If the direction of the axis seems to be understandable for *Sycon* with a single osculum, how is the axis directed in the polyoscular asconoid *Leucosolenia*? Considering tissue plasticity and physiology, it seems logical to assume that the axis has a functional nature, i.e., it is co-directional with the water flow passing through the body. This assumption is equally supported by data on the expression of Wnt in the osculum. In this regard, it seems promising to search for molecules other than Wnt involved in patterning of adult sponge: as axis patterning of an adult sponge seems unusual for us, additional undescribed mechanisms could be involved in its specification. At the moment, it is hard to interpret regeneration axial patterning data until molecular mechanisms of adult sponge patterning are revealed in different sponge classes.

However, if *wnt* is expressed only in the osculum (and the bases of the choanocyte chambers in *Sycon*), then how is the new axis of the regenerate established if there is no fragment of the osculum in it? We assume three possible options. Firstly, the leading role of Wnt was demonstrated only in the late stages of the axis formation during WBR [101]. Only one of *S. ciliatum wnt* is expressed by the first day after dissociation, and there are no data about the spatial pattern of this expression. Therefore, we suppose that Wnt could be an essential but not the first element in the signaling chain during axis formation. 

Secondly, the repertoire of Wnt components is exceptionally diverse. For example, the number of *wnt* in sponges is comparable to that in vertebrates, and the data of phylogenetic analysis indicate their independent emergence in evolution [145,147]. In addition, some of the essential elements of the signaling cascade, often in the shade, are extracellular inhibitors that bind the ligand, such as Dikkopf, WIF, Cerberus. Explicit orthologs of these proteins were not found in sponges; however, their interaction with the ligand underlined by cysteine-rich domains (CRD) and CRD-containing proteins, are widely represented in the sponge genome. Moreover, an additional level of regulation, inhibition of extracellular signal transmittance with CRD-containing proteins, could be involved. Thus, patterning of a new axis of a regenerate can occur due to the complex spatio-temporal expression of different Wnt components instead of a “constant model,” which postulated that *wnt* are permanently expressed in an osculum. However, current data available of Wnt pathway activity regulation are restricted to RNA-seq, in situ hybridization, and pharmacological inhibition analysis.

Finally, the system of local autoactivation-lateral inhibition (LALI), based on the Turing reaction-diffusion model [148,149], could be involved in the establishment of a new axis during WBR. This system is described to participate in establishing a pattern (for example, axial) in a system with uniform distribution of morphogen and its inhibitor (for example, wnt and sFRP), when the rate of wnt production depends on the sFRP, and vice versa. The functioning of this system was experimentally demonstrated on limb development, where TGF-beta is an activator of chondrogenesis and Noggin is an inhibitor, as well as on several other models [150]. The LALI can explain how asymmetry forms in radial symmetric primmorph with uniform distribution cells expressing, for example, wnt and its inhibitor. We suppose that one of the described mechanisms (or all of them) could be involved in axial patterning of a regenerate during WBR in sponges. Despite extensive research, many fundamental questions regarding the molecular mechanisms of the formation and maintenance of the body axis in sponge regeneration remain largely open because of limited research tools available for sponges. While we have rather broad data about gene diversity and expression across Porifera classes due to the expansion of DNA-, RNA-seq, as well as in situ hybridization, immunolocalization, and pharmacological assays, methods to study gene function are currently restricted to RNAi in two demosponges (Table S1).

Another significant gap in our knowledge concerns the diversity of developmental mechanisms underlying regeneration in Porifera. The available data suggest that cellular mechanisms are different across sponge classes but are stable within each class. In Calcarea and Homoscleromorpha, regeneration occurs through epithelial morphogeneses, e.g., spreading and fusion of the exopinacoderm and choanoderm during the formation of the regenerative membrane [36,38]. In demosponges, on the contrary, regeneration utilizes mesenchymal morphogeneses: migrations of individual cells, epithelial-to-mesenchymal, and mesenchymal-to-epithelial transitions [37,50]. Similar differences are also characteristic of morphogeneses accompanied development during sexual and asexual reproduction of these sponge clades [151]. 

Abundant cell dedifferentiations and transdifferentiations are commonly involved in regeneration processes across all sponge classes. At least in some cases, regeneration processes require multipotent cells, e.g., in the course WBR from small body fragments in some demosponges, when fragments are failing to regenerate if they consist of tissues devoid of archaeocytes and choanocytes [62,65,77,78]. However, pluripotent stem cells involved in WBR in investigated sponge classes are different: choanocytes and pinacocytes in calcareous sponges, and archaeocytes and choanocytes in demosponges. The cause of this trend should be further investigated using molecular tools.

The absence of robust knowledge of the fine mechanisms of the regeneration, their origin, and evolution highlight a demand in model sponge species for regeneration studies. Sponges are of particular importance for such evolutionary evaluations due to their basal phylogenetic position and high diversity of regenerative processes: physiological and reparative regeneration [6], various WBRs, including somatic embryogenesis. Considering the difference in cellular and morphogenetic mechanisms of regeneration across sponges, it is natural to develop several model species from distant phylogenetic clades and with the different anatomical organizations. Possible candidates species are *Leucosolenia variabilis* (Calcarea) [38,52,68,73], *Halisarca dujardinii* (Demospongiae) [41,45,50,75,145], and *Oscarella lobularis* (Homoscleromorpha) [36,84,152,153]. They are phylogenetically and morphologically distinct, have been already subjected to versatile experimental manipulations [84,153,154,155] (Table S1), and have the well-described morphogenetic and cellular basis of their reparative regeneration and some types of WBR.

Elaboration and detailed studies of sponge model species are very promising. Different types of WBR can be achieved after various operations in a single species. At the same time, these WBRs will share a common morphogenetic and cellular basis, requiring detailed morphological studies only once. These will make model species highly accessible for comprehensive physiological, biochemical, and molecular studies of regenerative mechanisms, as well as mechanisms of sponge body patterning.

## Supplementary Materials

The following are available online at https://www.mdpi.com/article/10.3390/genes12040506/s1, Table S1: Availability of molecular tools across Porifera classes.
