# Supplementary material for: Whole-Body Regeneration in Sponges: Diversity, Fine Mechanisms, and Future Prospects
Source: Genes (Basel). 2021 Mar 29;12(4):506. doi: 10.3390/genes12040506 (PMC8066720; doi:10.3390/genes12040506)
Supplement: Supplementary file 1 [file genes-12-00506-s001.pdf]

Supplementary Table 1. Availability of molecular tools across Porifera classes.

Class	Species	Long- term laboratory cultivation	Embryonic development studies	Regeneration studies	Genome	Transcriptome	Single-cell transcriptomes	Immunolocalization	*In situ* hybridization	Pharmacological assay	Transfection	RNAi	References
**Calcarea**	*Sycon ciliatum*	–	●	○	●	●	×	×	●	×	×	×	[1–7]
	Leucosolenia complicata	–	●	●	●	●	×	×	●	×	×	×	[4,6,8–16]
	Leucosolenia cf. variabilis	±	○	●	○	○	×	●	○	○	×	×	[17,18]
**Homoscleromorpha**	*Oscarella lobularis*	±	●	●	○	●	○	●	●	●	○	×	[19–28]
	Oscarella pearsei	–	○	○	●	●	×	●	×	×	×	×	[29–31]
**Demospongiae**	*Amphimedon queenslandica*	±	●	×	●	●	●	●	●	●	○	×	[7,22,26,32–42]
	Ephydatia fluviatilis	±	●	●	×	●	●	×	●	●	×	×	[43–51]
	Ephydatia muelleri	±	×	×	●	●	×	●	●	●	●	●	[52–60]
	Spongilla lacustris	±	●	●	×	●	×	●	×	●	●	×	[43,61–67]
	Halisarca caerulea	±	×	●	×	●	×	●	×	×	×	×	[68,69]
	Halisarca dujardinii	±	●	●	○	●	×	●	●	○	×	×	[70–82]
	Tethya wilhelma	+	×	×	●	●	×	×	×	×	●	●	[58,83]
	Suberites domuncula	+	×	●	×	×	×	×	×	●	●	×	[84–86]
**Hexactinellida**	*Aphrocallistes vastus*	–	×	×	×	●	×	×	×	×	×	×	[61,62,87]
	Oopsacas minuta	–	●	×	×	●	×	×	×	×	×	×	[87–90]

Long-term laboratory cultivation: + available, ± restrictedly available, – unavailable. Developmental data and molecular tools: ● available, ○ in progress, × unavailable.

References

1. Fortunato, S.A.V.; Adamski, M.; Bergum, B.; Guder, C.; Jordal, S.; Leininger, S.; Zwafink, C.; Rapp, H.T.; Adamska, M. Genome-wide analysis of the sox family in the calcareous sponge *Sycon ciliatum*: multiple genes with unique expression patterns. *Evodevo* **2012**, *3*, 14, doi:10.1186/2041-9139-3-14.

2. Laplante, M.; Adamska, M.; Leininger, S.; Ereskovsky, A.V. *Sycon ciliatum* (Calcarea, Calcaronea) regeneration peculiarities. In Proceedings of the 5th meeting of the European Society for Evolutionary Developmental Biology; 2014; pp. 374–375.

3. Soubigou, A.; Ross, E.G.; Touhami, Y.; Chrismas, N.; Modepalli, V. Regeneration in the sponge *Sycon ciliatum* partly mimics postlarval development. *Development* **2020**, *147*, dev193714, doi:10.1242/dev.193714.

4. Fortunato, S.A.V.; Adamski, M.; Adamska, M. Comparative analyses of developmental transcription factor repertoires in sponges reveal unexpected complexity of the earliest animals. *Mar. Genomics* **2015**, *24*, 121–129, doi:10.1016/j.margen.2015.07.008.

5. Leininger, S.; Adamski, M.; Bergum, B.; Guder, C.; Liu, J.; Laplante, M.; Bråte, J.; Hoffmann, F.; Fortunato, S.A.V.; Jordal, S.; et al. Developmental gene expression provides clues to relationships between sponge and eumetazoan body plans. *Nat. Commun.* **2014**, *5*, 3905, doi:10.1038/ncomms4905.

6. Voigt, O.; Adamski, M.; Sluzek, K.; Adamska, M. Calcareous sponge genomes reveal complex evolution of α-carbonic anhydrases and two key biomineralization enzymes. *BMC Evol. Biol.* **2014**, *14*, 230, doi:10.1186/s12862-014-0230-z.

7. Degnan, B.M.; Adamska, M.; Richards, G.S.; Larroux, C.; Leininger, S.; Bergum, B.; Calcino, A.; Taylor, K.; Nakanishi, N.; Degnan, S.M. Porifera. In *Evolutionary Developmental Biology of Invertebrates 1: Introduction, Non-Bilateria, Acoelomorpha, Xenoturbellida, Chaetognatha*; Wanninger, A., Ed.; Springer-Verlag: Wien, 2015; pp. 65–106.

8. Anakina, R.P. The embryological development of Barents Sea sponge *Leucosolenia complicata* Mont. (Calcarea). In *Morphogenesis in sponges*; Korotkova, G.P., Ed.; Leningrad University Press: Leningrad, 1981; pp. 52–58.

9. Anakina, R.P. The Cleavage specifity in Embryoes of the Barents Sea Sponge *Leucosolenia complicata* Montagu (Calcispongiae, Calcaronea). In *Modern Problems of Poriferan Biology*; Ereskovsky, A.V., Keupp, H., Kohring, R., Eds.; Berliner Geowiss Abh, Freie University: Berlin, 1997; pp. 45–53 ISBN 3--496--00365--0.

10. Korotkova, G.P. Regeneration and cellular proliferation in calcareous sponge *Leucosolenia complicata* Mont. *Vestn. Leningr. Univ.* **1961**, *4*, 39–50.

11. Korotkova, G.P. Peculiarities of morphogenesis during the development of the calcareous sponge *Leucosolenia complicata* Mont. from a small pieces of the body wall. *Vestn. Leningr. Univ.* **1969**, *15*, 15–22.

12. Korotkova, G.P. Comparative morphological investigations of development of sponges from dissociated cells. *Trans. Leningr. Soc. Nat.* **1972**, *78*, 74–109.

13. Korotkova, G.P.; Gelihovskaia, M.A. Recherches Experimentales sur le Phenomene de Polarite Chez les Eponges Calcaires du Type Ascon. *Cah. Biol. Mar.* **1963**, *4*, 47–60.

14. Jones, W.C. The Contractility and Healing Behaviour of Pieces of *Leucosotenia complicata*. *Q. J. Microsc. Sci.* **1957**, *98*, 203–217.

15. Korotkova, G.P. Behaviour of the cellular elements in the calcareous sponge *Leucosolenia complicata* Mont. during regeneration. *Acta Biol. Acad. Sci. Hung.* **1962**, *13*, 1–30.

16. Fortunato, S.A.V.; Adamski, M.; Ramos, O.M.; Leininger, S.; Liu, J.; Ferrier, D.E.K.; Adamska, M. Calcisponges have a ParaHox gene and dynamic expression of dispersed NK homeobox genes. *Nature* **2014**, *514*, 620–623, doi:10.1038/nature13881.

17. Ereskovsky, A.V.; Lavrov, A.I.; Bolshakov, F.V.; Tokina, D.B. Regeneration in White Sea sponge *Leucosolenia complicata* (Porifera, Calcarea). *Invertebr. Zool.* **2017**, *14*, 108–113, doi:10.15298/invertzool.14.2.02.

18. Lavrov, A.I.; Bolshakov, F.V.; Tokina, D.B.; Ereskovsky, A.V. Sewing up the wounds: The epithelial morphogenesis as a central mechanism of calcaronean sponge regeneration. *J. Exp. Zool. Part B Mol. Dev. Evol.* **2018**, *330*, 351–371, doi:10.1002/jez.b.22830.

19. Lapébie, P.; Gazave, E.; Ereskovsky, A.V.; Derelle, R.; Bézac, C.; Renard, E.; Houliston, E.; Borchiellini, C. WNT/beta-catenin signalling and epithelial patterning in the homoscleromorph sponge *Oscarella*. *PLoS One* **2009**, *4*, e5823, doi:10.1371/journal.pone.0005823.

20. Fierro-Constaín, L.; Schenkelaars, Q.; Gazave, E.; Haguenauer, A.; Rocher, C.; Ereskovsky, A.V.; Borchiellini, C.; Renard, E. The Conservation of the Germline Multipotency Program, from Sponges to Vertebrates: A Stepping Stone to Understanding the Somatic and Germline Origins. *Genome Biol. Evol.* **2017**, *9*, evw289, doi:10.1093/gbe/evw289.

21. Ereskovsky, A.V.; Borisenko, I.E.; Lapébie, P.; Gazave, E.; Tokina, D.B.; Borchiellini, C. *Oscarella lobularis* (Homoscleromorpha, Porifera) Regeneration: Epithelial Morphogenesis and Metaplasia. *PLoS One* **2015**, *10*, e0134566, doi:10.1371/journal.pone.0134566.

22. Rocher, C.; Chenesseau, S.; Marschal, M.; Le Goff, E.; Dutilleul, M.; Marschal, F.; Massey-Harroche, D.; Ereskovsky, A.V.; Borchiellini, C.; Renard, E. The buds of *Oscarella lobularis* (Porifera): a new convenient model for sponge cell and developmental biology. *bioRxiv* **2020**.

23. Ereskovsky, A.V.; Tokina, D.B. Asexual reproduction in homoscleromorph sponges (Porifera; Homoscleromorpha). *Mar. Biol.* **2007**, *151*, 425–434, doi:10.1007/s00227-006-0439-5.

24. Ereskovsky, A.V.; Boury-Esnault, N. Cleavage pattern in *Oscarella* species (Porifera, Demospongiae, Homoscleromorpha): transmission of maternal cells and symbiotic bacteria. *J. Nat. Hist.* **2002**, *36*, 1761–1775.

25. Boury-Esnault, N.; Ereskovsky, A.V.; Bezac, C.; Tokina, D.B. Larval development in the Homoscleromorpha (Porifera, Demospongiae). *Invertebr. Biol.* **2003**, *122*, 187–202.

26. Borchiellini, C.; Degnan, S.M.; Le Goff, E.; Rocher, C.; Baghdiguian, S.; Séjourné, N.; Marschal, F.; Le Bivic, A.; Godefroy, N.; Degnan, B.M.; et al. Staining and Tracking Methods for Studying Sponge Cell Dynamics. In *Developmental Biology of the Sea Urchin and Other Marine Invertebrates: Methods and Protocols*; Caroll, D.J., Stricker, S.A., Eds.; Springer, 2021; pp. 81–97 ISBN 9781071609743.

27. Fierro-Constaín, L.; Rocher, C.; Marschal, F.; Schenkelaars, Q.; Séjourné, N.; Borchiellini, C.; Renard, E. In Situ Hybridization Techniques in the Homoscleromorph Sponge *Oscarella lobularis*. In *Developmental Biology of the Sea Urchin and Other Marine Invertebrates: Methods and Protocols*; Caroll, D.J., Stricker, S.A., Eds.; Springer, 2021; pp. 181–194.

28. Vernale, A.; Prünster, M.M.; Marchianó, F.; Debost, H.; Brouilly, N.; Rocher, C.; Massey-Harroche, D.; Renard, E.; Le Bivic, A.; Habermann, B.H.; et al. Evolution of mechanisms controlling epithelial morphogenesis across animals: new insights from dissociation - reaggregation experiments in the sponge *Oscarella lobularis*. *bioRxiv* **2021**, 1–71, doi:10.1101/2021.03.22.436370.

29. Nichols, S.A.; Roberts, B.W.W.; Richter, D.J.J.; Fairclough, S.R.; King, N. Origin of metazoan cadherin diversity and the antiquity of the classical cadherin/β-catenin complex. *Proc. Natl. Acad. Sci.* **2012**, *109*, 13046–13051, doi:10.1073/pnas.1120685109.

30. Ereskovsky, A.V.; Richter, D.J.; Lavrov, D.V.; Schippers, K.J.; Nichols, S.A. Transcriptome sequencing and delimitation of sympatric *Oscarella* species (O. carmela and O. pearsei sp. nov) from California, USA. *PLoS One* **2017**, *12*, e0183002, doi:10.1371/journal.pone.0183002.

31. Miller, P.W.; Pokutta, S.; Mitchell, J.M.; Chodaparambil, J.V.; Clarke, D.N.; Nelson, W.J.; Weis, W.I.; Nichols, S.A. Analysis of a vinculin homolog in a sponge (phylum Porifera) reveals that vertebrate-like cell adhesions emerged early in animal evolution. *J. Biol. Chem.* **2018**, *293*, 11674–11686, doi:10.1074/jbc.RA117.001325.

32. Sogabe, S. The biology of choanocytes and choanocyte chambers and their role in the sponge stem cell system, The University of Queensland, 2017.

33. Ueda, N.; Richards, G.S.; Degnan, B.M.; Kranz, A.; Adamska, M.; Croll, R.P.; Degnan, S.M. An ancient role for nitric oxide in regulating the animal pelagobenthic life cycle: evidence from a marine sponge. *Sci. Rep.* **2016**, *6*, 37546, doi:10.1038/srep37546.

34. Sogabe, S.; Nakanishi, N.; Degnan, B.M. The ontogeny of choanocyte chambers during metamorphosis in the demosponge *Amphimedon queenslandica*. *Evodevo* **2016**, *7*, 1–13, doi:10.1186/s13227-016-0042-x.

35. Fernandez-Valverde, S.L.; Calcino, A.D.; Degnan, B.M. Deep developmental transcriptome sequencing uncovers numerous new genes and enhances gene annotation in the sponge *Amphimedon queenslandica*. *BMC Genomics* **2015**, *16*, 387, doi:10.1186/s12864-015-1588-z.

36. Srivastava, M.; Simakov, O.; Chapman, J.; Fahey, B.; Gauthier, M.E.A.; Mitros, T.; Richards, G.S.; Conaco, C.; Dacre, M.; Hellsten, U.; et al.

The *Amphimedon queenslandica* genome and the evolution of animal complexity. *Nature* **2010**, *466*, 720–6, doi:10.1038/nature09201.

37. Grice, L.F.; Bernard, C.; Degnan, B.M. Transcriptomic Profiling of the Allorecognition Response to Grafting in the Demosponge *Amphimedon queenslandica*. *Mar. Drugs* **2017**, *15*, 136, doi:10.3390/md15050136.

38. Adamska, M.; Degnan, S.M.; Green, K.M.; Adamski, M.; Craigie, A.; Larroux, C.; Degnan, B.M. Wnt and TGF-beta expression in the sponge Amphimedon queenslandica and the origin of metazoan embryonic patterning. *PLoS One* **2007**, *2*, e1031, doi:10.1371/journal.pone.0001031.

39. Adamska, M.; Larroux, C.; Adamski, M.; Green, K.M.; Lovas, E.; Koop, D.; Richards, G.S.; Zwafink, C.; Degnan, B.M. Structure and expression of conserved Wnt pathway components in the demosponge *Amphimedon queenslandica*. *Evol. Dev.* **2010**, *12*, 494–518, doi:10.1111/j.1525-142X.2010.00435.x.

40. Sogabe, S.; Hatleberg, W.L.; Kocot, K.M.; Say, T.E.; Stoupin, D.; Roper, K.E.; Fernandez-Valverde, S.L.; Degnan, S.M.; Degnan, B.M. Pluripotency and the origin of animal multicellularity. *Nature* **2019**, *570*, 519–522, doi:10.1038/s41586-019-1290-4.

41. Larroux, C.; Fahey, B.; Adamska, M.; Richards, G.S.; Gauthier, M.; Green, K.M.; Lovas, E.; Degnan, B.M. Whole-mount in situ hybridization in *Amphimedon*. *Cold Spring Harb. Protoc.* **2008**, *3*, 1–9, doi:10.1101/pdb.prot5096.

42. Sebé-Pedrós, A.; Chomsky, E.; Pang, K.; Lara-Astiaso, D.; Gaiti, F.; Mukamel, Z.; Amit, I.; Hejnol, A.; Degnan, B.M.; Tanay, A. Early metazoan cell type diversity and the evolution of multicellular gene regulation. *Nat. Ecol. Evol.* **2018**, *2*, 1176–1188, doi:10.1038/s41559-018-0575-6.

43. Efremova, S.M. Morphophysiological analysis of the development of freshwater sponges *Ephydatia fluviatilis* and *Spongilla lacustris* from dissociated cells. *Trans. Leningr. Soc. Nat.* **1972**, *78*, 110–154.

44. Funayama, N.; Nakatsukasa, M.; Hayashi, T.; Agata, K. Isolation of the choanocyte in the fresh water sponge, *Ephydatia fluviatilis* and its lineage marker, Ef annexin. *Dev. Growth Differ.* **2005**, *47*, 243–53, doi:10.1111/j.1440-169X.2005.00800.x.

45. Nakayama, S.; Arima, K.; Kawai, K.; Mohri, K.; Inui, C.; Sugano, W.; Koba, H.; Tamada, K.; Nakata, Y.J.; Kishimoto, K.; et al. Dynamic Transport and Cementation of Skeletal Elements Build Up the Pole-and-Beam Structured Skeleton of Sponges. *Curr. Biol.* **2015**, 1–6, doi:10.1016/j.cub.2015.08.023.

46. Alié, A.; Hayashi, T.; Sugimura, I.; Manuel, M.; Sugano, W.; Mano, A.; Satoh, N.; Agata, K.; Funayama, N. The ancestral gene repertoire of animal stem cells. *Proc. Natl. Acad. Sci.* **2015**, 201514789, doi:10.1073/pnas.1514789112.

47. Sukhodolskaya, A.N. Mechanisms of oscular tube regeneration in *Ephydatia fluviatilis* (L.). In *Morphogenetic processes during asexual reproduction, somatic embryogenesis and regeneration*; Tokin, B.P., Ed.; Leningrad University Publishing House, 1973; pp. 127–146.

48. Sukhodolskaya, A.N.; Stoliarov, A.M. Peculiarites of development of *Ephydatia fluviatilis* from small body fragment. *Vestn. Leningr. Univ.* **1974**, *15*, 12–19.

49. Brien, P. La réorganisation de l'éponge après dissociation par filtration et phénomènes d'involution chez *Ephydatia fluviatilis*. *Arch. Biol. (Liege).* **1937**, *48*, 185–268.

50. Saller, U. Oogenesis and larval development of *Ephydatia fluviatilis* (Porifera, Spongillidae). *Zoomorphology* **1988**, *108*, 23–28, doi:10.1007/BF00312211.

51. Wielspütz, C.; Saller, U. The metamorphosis of the parenchymula-larva of *Ephydatia fluviatilis* (Porifera, Spongillidae). *Zoomorphology* **1990**, *109*, 173–177.

52. Peña, J.F.; Alié, A.; Richter, D.J.; Wang, L.; Funayama, N.; Nichols, S.A. Conserved expression of vertebrate microvillar gene homologs in choanocytes of freshwater sponges. *Evodevo* **2016**, *7*, 13, doi:10.1186/s13227-016-0050-x.

53. Schippers, K.J.; Nichols, S.A. Evidence of Signaling and Adhesion Roles for β-Catenin in the Sponge *Ephydatia muelleri*. *Mol. Biol. Evol.* **2018**, *35*, 1407–1421, doi:10.1093/molbev/msy033.

54. Mitchell, J.M.; Nichols, S.A. Diverse cell junctions with unique molecular composition in tissues of a sponge (Porifera). *Evodevo* **2019**, *10*, 26, doi:10.1186/s13227-019-0139-0.

55. Rivera, A.; Winters, I.; Rued, A.; Ding, S.; Posfai, D.; Cieniewicz, B.; Cameron, K.; Gentile, L.; Hill, A. The evolution and function of the Pax/Six regulatory network in sponges. *Evol. Dev.* **2013**, *15*, 186–196, doi:10.1111/ede.12032.

56. Hall, C.; Rodriguez, M.; Garcia, J.; Posfai, D.; DuMez, R.; Wictor, E.; Quintero, O.A.; Hill, M.S.; Rivera, A.S.; Hill, A.L. Secreted frizzled related protein is a target of PaxB and plays a role in aquiferous system development in the freshwater sponge, *Ephydatia muelleri*. *PLoS One* **2019**, *14*, e0212005, doi:10.1371/journal.pone.0212005.

57. Schenkelaars, Q.; Quintero, O.; Hall, C.; Fierro-Constain, L.; Renard, E.; Borchiellini, C.; Hill, A.L. ROCK inhibition abolishes the establishment of the aquiferous system in *Ephydatia muelleri* (Porifera, Demospongiae). *Dev. Biol.* **2016**, *412*, 298–310, doi:10.1016/j.ydbio.2016.02.026.

58. Rivera, A.S.; Hammel, J.U.; Haen, K.M.; Danka, E.S.; Cieniewicz, B.; Winters, I.P.; Posfai, D.; Wörheide, G.; Lavrov, D.V.; Knight, S.W.; et al. RNA interference in marine and freshwater sponges: actin knockdown in *Tethya wilhelma* and *Ephydatia muelleri* by ingested dsRNA expressing bacteria. *BMC Biotechnol.* **2011**, *11*, 67, doi:10.1186/1472-6750-11-67.

59. Windsor, P.J.; Leys, S.P. Wnt signaling and induction in the sponge aquiferous system: evidence for an ancient origin of the organizer. *Evol. Dev.* **2010**, *12*, 484–93, doi:10.1111/j.1525-142X.2010.00434.x.

60. Kenny, N.J.; Francis, W.R.; Rivera-Vicens, R.E.; Juravel, K.; de Mendoza, A.; Diez-Vives, C.; Lister, R.; Bezares-Calderon, L.; Grombacher, L.; Roller, M.; et al. The genomic basis of animal origins: a chromosomal perspective from the sponge *Ephydatia muelleri*. **2020**, 1–31.

61. Riesgo, A.; Farrar, N.; Windsor, P.J.; Giribet, G.; Leys, S.P. The analysis of eight transcriptomes from all poriferan classes reveals surprising genetic complexity in sponges. *Mol. Biol. Evol.* **2014**, *31*, 1102–20, doi:10.1093/molbev/msu057.

62. Windsor Reid, P.J.; Matveev, E.; McClymont, A.; Posfai, D.; Hill, A.L.; Leys, S.P. Wnt signaling and polarity in freshwater sponges. *BMC Evol. Biol.* **2018**, *18*, 12, doi:10.1186/s12862-018-1118-0.

63. Saller, U.; Weissenfels, N. The development of *Spongilla lacustris* from the oocyte to the free larva (Porifera, Spongillidae). *Zoomorphology* **1985**, *105*, 367–374.

64. Pfannkuchen, M.; Brümmer, F. Heterologous expression of DsRed2 in young sponges (Porifera). *Int. J. Dev. Biol.* **2009**, *53*, 1113–1117, doi:10.1387/ijdb.072526mp.

65. Wachtmann, D.; Stockem, W.; Weissenfels, N. Cytoskeletal organization and cell organelle transport in basal epithelial cells of the freshwater sponge *Spongilla lacustris*. *Cell Tissue Res.* **1990**, *261*, 145–154, doi:10.1007/BF00329447.

66. Wachtmann, D.; Stockem, W. Microtubule- and microfilament-based dynamic activities of the endoplasmic reticulum and the cell surface in epithelial cells of *Spongilla lacustris* (Porifera, Spongillidae). *Zoomorphology* **1992**, *112*, 117–124, doi:10.1007/BF01633102.

67. Sukhodolskaya, A.N.; Ivanova, L.V. Somatic embryogenesis of some spongillidae during reproductive period of their life cycle. *Arch. AGE* **1980**, *79*, 80–88.

68. Alexander, B.E.; Achlatis, M.; Osinga, R.; van der Geest, H.G.; Cleutjens, J.P.M.; Schutte, B.; de Goeij, J.M. Cell kinetics during regeneration in the sponge *Halisarca caerulea*: how local is the response to tissue damage? *PeerJ* **2015**, *3*, e820, doi:10.7717/peerj.820.

69. Kenny, N.J.; de Goeij, J.M.; de Bakker, D.M.; Whalen, C.G.; Berezikov, E.; Riesgo, A. Towards the identification of ancestrally shared regenerative mechanisms across the Metazoa: A Transcriptomic case study in the Demosponge *Halisarca caerulea*. *Mar. Genomics* **2017**, *44*, 1–35, doi:10.1016/j.margen.2017.11.001.

70. Korotkova, G.P.; Sukhodolskaya, A.N.; Krasukevitch, T.N. The pecularities of morphogenesis of the development of *Halisarca dujardini* from the small part of the body. *Vestn. Leningr. Univ.* **1983**, *9*, 41–46.

71. Volkova, M.A.; Zolotareva, G.A. The development of *Halisarca dujardini* Johnston from conglomerates of somatic cells. In *Morphogenesis in sponges*; Korotkova, G.P., Ed.; Leningrad University Press: Leninrad, 1981; pp. 74–93.

72. Borisenko, I.E.; Adamski, M.; Ereskovsky, A.V.; Adamska, M. Surprisingly rich repertoire of Wnt genes in the demosponge *Halisarca*

dujardini. *BMC Evol. Biol.* **2016**, *16*, 123, doi:10.1186/s12862-016-0700-6.

73. Mukhina, Y.I.; Kumeiko, V. V.; Podgornaya, O.I.; Efremova, S.M. The fate of larval flagellated cells during metamorphosis of the sponge *Halisarca dujardini*. *Int. J. Dev. Biol.* **2006**, *50*, 533–41, doi:10.1387/ijdb.052123ym.

74. Mukhina, Y.I.; Kumeiko, V. V.; Podgornaya, O.I.; Efremova, S.M. The events of metamorphosis in the demosponge *Halisarca dujardini* Johnston , 1842 , studied with immunocytochemical method. In *Porifera Research: Biodiversity, Innovation and Sustainability*; Custódio, M.R., Lôbo-Hajdu, G., Hajdu, E., Muricy, G., Eds.; Museu Nacional: Rio De Jeneiro, 2007; pp. 483–490.

75. Lavrov, A.I.; Kosevich, I.A. Sponge cell reaggregation: Cellular structure and morphogenetic potencies of multicellular aggregates. *J. Exp. Zool. Part A Ecol. Genet. Physiol.* **2016**, *325*, 158–177, doi:10.1002/jez.2006.

76. Borisenko, I.E.; Adamska, M.; Tokina, D.B.; Ereskovsky, A.V. Transdifferentiation is a driving force of regeneration in *Halisarca dujardini* (Demospongiae, Porifera). *PeerJ* **2015**, *3*, e1211, doi:10.7717/peerj.1211.

77. Gonobobleva, E.L.; Ereskovsky, A.V. Metamorphosis of the larva of *Halisarca dujardini* (Demospongiae, Halisarcida). *Bull. Inst. R. Sci. Nat. Belg* **2004**, *74*, 101–115.

78. Ereskovsky, A.V.; Konjukov, P.; Willenz, P. Experimental Metamorphosis of *Halisarca dujardini* Larvae (Demospongiae , Halisarcida): Evidence of Flagellated Cell Totipotentiality. *J. Morphol.* **2007**, *536*, 529–536, doi:10.1002/jmor.

79. Gonobobleva, E.L.; Ereskovsky, A.V. Polymorphism in free-swimming larvae of *Halisarca dujardini* (Demospongiae, Halisarcida). *Boll. Mus. Ist. Biol. Univ. Genova* **2004**, *68*, 349–356.

80. Ereskovsky, A.V.; Gonobobleva, E.L.; Vishnyakov, A. Morphological evidence for vertical transmission of symbiotic bacteria in the viviparous sponge *Halisarca dujardini* Johnston (Porifera, Demospongiae, Halisarcida). *Mar. Biol.* **2005**, *146*, 869–875, doi:10.1007/s00227-004-1489-1.

81. Ereskovsky, A.V.; Gonobobleva, E.L. New data on embryonic development of *Halisarca dujardini* Johnston, 1842 (Demospongiae , Halisarcida). *Zoosystema* **2000**, *1842*, 355–368.

82. Ereskovsky, A.V.; Gonobobleva, E.L. Development of *Halisarca dujardini* Johnston 1842 (Porifera, Ceractinomorpha: Halisarcida) from egg to free larva. In *Memoirs of the Queensland Museum*; 1999; Vol. 44, pp. 598–603 ISBN 0787940690.

83. Francis, W.R.; Eitel, M.; Vargas, S.; Adamski, M.; Haddock, S.H.D.; Krebs, S.; Blum, H.; Erpenbeck, D.; Wörheide, G. The genome of the contractile demosponge *Tethya wilhelma* and the evolution of metazoan neural signalling pathways. *bioRxi* **2017**, doi:10.1101/120998.

84. Custódio, M.R.; Prokic, I.; Steffen, R.; Koziol, C.; Borojevic, R.; Brümmer, F.; Nickel, M.; Müller, W.E.. Primmorphs generated from dissociated cells of the sponge *Suberites domuncula*: a model system for studies of cell proliferation and cell death. *Mech. Ageing Dev.* **1998**,

105, 45–59.

85. Sipkema, D.; van Wielink, R.; van Lammeren, A.A.M.; Tramper, J.; Osinga, R.; Wijffels, R.H. Primmorphs from seven marine sponges: formation and structure. *J. Biotechnol.* **2003**, *100*, 127–39.

86. Revilla-i-Domingo, R.; Schmidt, C.; Zifko, C.; Raible, F. Establishment of Transgenesis in the Demosponge *Suberites domuncula*. *Genetics* **2018**, *210*, genetics-301121, doi:10.1534/genetics.118.301121.

87. Schenkelaars, Q.; Pratlong, M.; Kodjabachian, L.; Fierro-, L.; Vacelet, J.; Bivic, A.L.; Renard, E.; Borchiellini, C. Animal multicellularity and polarity without Wnt signaling. *Sci. Rep.* **2017**, *7*, 15383, doi:10.1038/s41598-017-15557-5.

88. Leys, S.P. Embryogenesis in the glass sponge *Oopsacas minuta*: Formation of syncytia by fusion of blastomeres. *Integr. Comp. Biol.* **2006**, *46*, 104–117, doi:10.1093/icb/icj016.

89. Boury-Esnault, N.; Vacelet, J. Preliminary studies on the organization and development of a hexactinellid sponge from a Mediterranean cave, *Oopsacas minuta*. In *Sponges in time and space: Biology, chemistry, paleontology*; van Soest, R.W.M., van Kempen, T.M.G., Braekman, J.-C., Eds.; A.A. Balkema: Rotterdam, 1994; pp. 407–415.

90. Boury-Esnault, N.; Efremova, S.M.; Bézac, C.; Vacelet, J. Reproduction of a hexactinellid sponge: first description of gastrulation by cellular delamination in the Porifera. *Invertebr. Reprod. Dev.* **1999**, *35*, 187–201.
